# Bacteremia from streptococcus constellatus revealing a gastrointestinal stromal tumor

**DOI:** 10.1186/s13099-023-00593-6

**Published:** 2024-01-04

**Authors:** Salvatore Chessa, Elena Belfiori, Giulia Mandis, Enrico Urru, Giovanna Manconi, Angelo Scuteri

**Affiliations:** 1 Post-Graduate Medical School of Internal Medicine, University of Cagliari, Cagliari, Italy; 2 Department of Medical Sciences and Public Health, University of Cagliari, Cagliari, Italy; 3 Division of Internal Medicine, University Hospital Monserrato (AOU), Cagliari, Italy

**Keywords:** *Streptococcus constellatus*, Bacteriemia, Pyogenic liver abscesses, GIST

## Abstract

**Background:**

Pyogenic Liver Abscesses (PLA) are the most common type of visceral abscess. They generally develop in a context of biliary disease or hematogenous seeding, but a complete diagnostic work-up is always required in order not to miss other important causes, including above all malignancies of the gastro-intestinal tract.

**Case presentation:**

Herein, we report a particular case of a 80 years-old immunocompetent woman hospitalized for sepsis. At the end of the diagnostic process, *Streptococcus constellatus* (*Sc)* was identified as the cause of sepsis, multiple PLA were found together with a previous unknown ileal malignancy. We speculated about a possible correlation among these three entities (i.e. sepsis from *Sc*, PLA and tumors).

**Conclusions:**

Detection of *Sc* in blood should raise red flags in clinicians as aggressive clinical presentation are possible.

## Background

*Sc* is usually part of the normal flora of mouth and gastro-intestinal tract, but aggressive presentations are possible. They mainly consist in abscesses formation and this can occur in association with malignancy [1].

## Case presentation

An 80-year-old Caucasian woman, with a medical history of hypertensive heart disease, reached the emergency room (ER) because of a fever lasting 3 days occurring after dental treatment, and no other relevant symptoms.

She also reported two hospitalizations over the previous year: one for anaemia and unexplained deep vein thrombosis of the right leg; the other one for bowel sub-occlusion. During these previous admissions, an abdominal CT scan detected the presence of a jejunal mass, described as a mixed (solid-liquid) roundish lesion (54 × 48 mm). The surgeon did not prescribe any additional diagnostic investigation of the mass.

At the admission to the ER, the patient was febrile (38,5 °C). No remarkable signs were observable at physical examination and chest X-ray was negative. Blood test showed leucocytosis (23.6 × 10^9^/L) with 84.4% neutrophils and increase of both CRP (88.2 mg/L, normal values – nv − 0.0–5.0) and procalcitonin (41.2 ng/ml, nv < 0.5). After blood sample was drawn for blood cultures, an empirical antibiotic therapy with piperacillin-tazobactam was initiated.

Thereafter, the patient was transferred to our Internal Medicine Unit.

On the 3rd day an hypoxemic respiratory failure occurred, despite no inflammation nor infective process was observable on CT scan. It was interpreted as the first manifestation of a probable incipient Multiple Organ Dysfunction Syndrome (MODS). Blood tests only slightly improved (14.3 × 10^9^/L leucocytes with 76% neutrophils, CRP 111.9 mg/L, and procalcitonin 1.8 ng/mL).

On day 6th blood cultures showed the growth of *Sc*.

Strains of *Sc* were identified in the blood by *matrix-assisted laser desorption/ ionization time-of-flight mass spectrometry* (MALDI-TOF) using the VITEK® MS system (bioMérieux).

The pathogen showed the following antibiotic resistance profile:

**Table 1:** Antibiotic resistance profile determined from blood cultures

ANTHIBIOTIC		MIC
Ampicillin	S	< 0,25
Cefotaxime	S	0,5
Ceftriaxone	S	
Clindamycin	R	
Linezolid	(-	
Levofloxacin	(-	
Penicillin G	I	
Teicoplanin	S	
Vancomycin	S	0.25

Sensitivity to piperacillin-tazobactam was not part of the routine test of our Laboratory. After the lack of a significant improvement in clinical or laboratory parameters, teicoplanin was added to therapy and, after four days of treatment, produced a relevant benefit on respiratory signs and oxygen supplementation demand, as well as on laboratory test (further decrease of WBC to 11.3 × 10^9^/L leucocytes with 67% neutrophils, CRP 17.5 mg/L, and normalization of procalcitonin).

Meanwhile, we searched for septic foci. Urine culture was negative. A CT scan of facial bones did not show any periodontal inflammation or pulpal infection, ruling out the oral cavity as the source of the bacteremia. Unfortunately, oral cavity swabbing was not performed, thus precluding the possibility to ascertain whether the same *Sc* was also part of the “normal flora” of our patient.

**Fig. 1:**
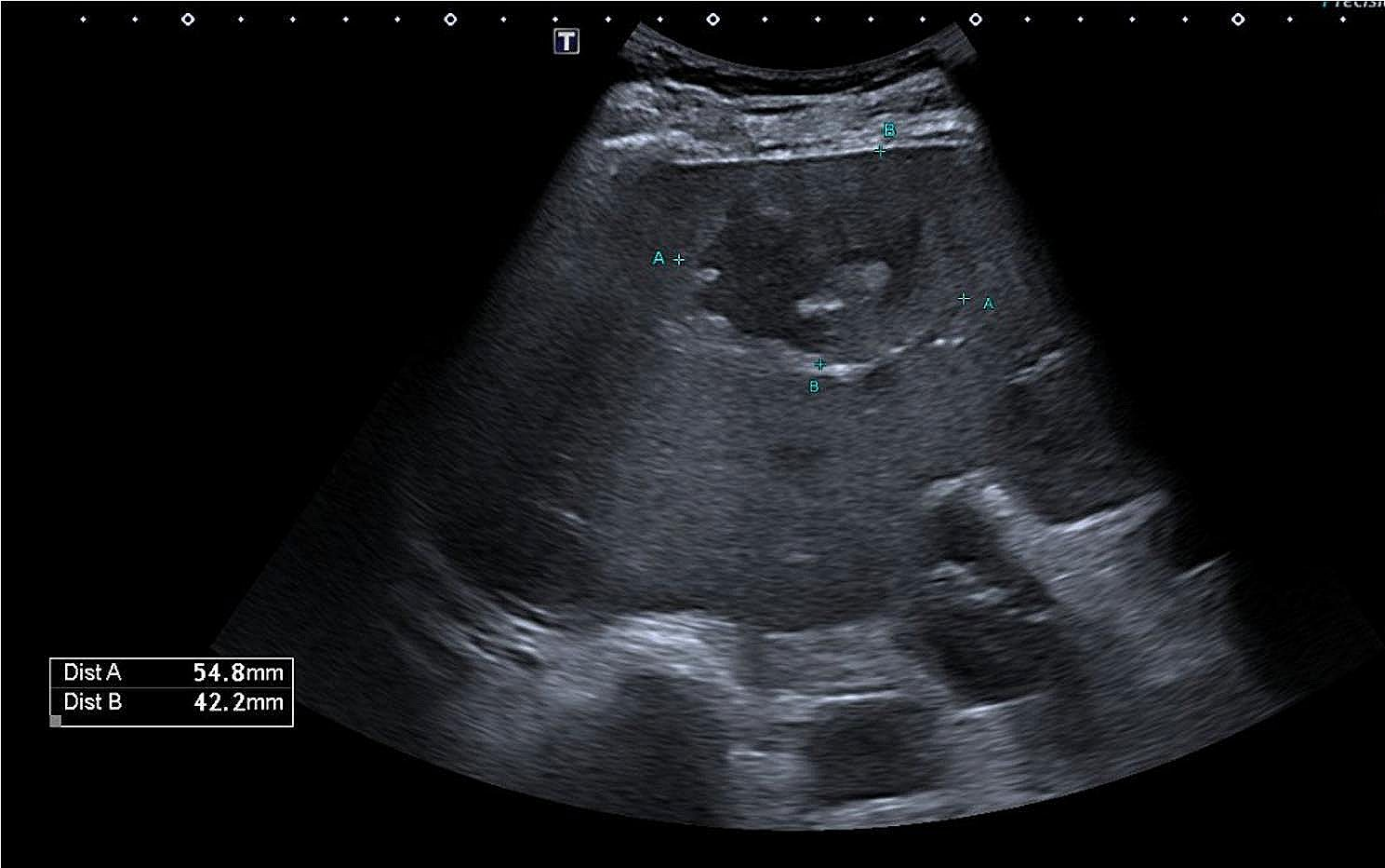
Abdominal US of the liver showing, in SVIII, a roundish, poorly demarcated lesion of 54.8 × 42.2 mm in size. It shows a dishomogeneous appearance as it mainly consists of a hypoechoic area, suggestive for colliquation, with some internal echoes. The differential diagnosis of a focal liver lesion like this is wide, including primitive or metastatic lesions, and clinical correlation is essential to guide the diagnostic process. Second-line methods such as contrast-enhanced ultrasonography (CEUS), computed tomography (CT) or magnetic resonance (MR) are imperative in these cases and help to correctly determine the nature of these lesions

Echocardiography excluded an infective endocarditis. An abdominal US revealed the presence of multiple hepatic lesions (Fig. 1).

**Fig. 2:**
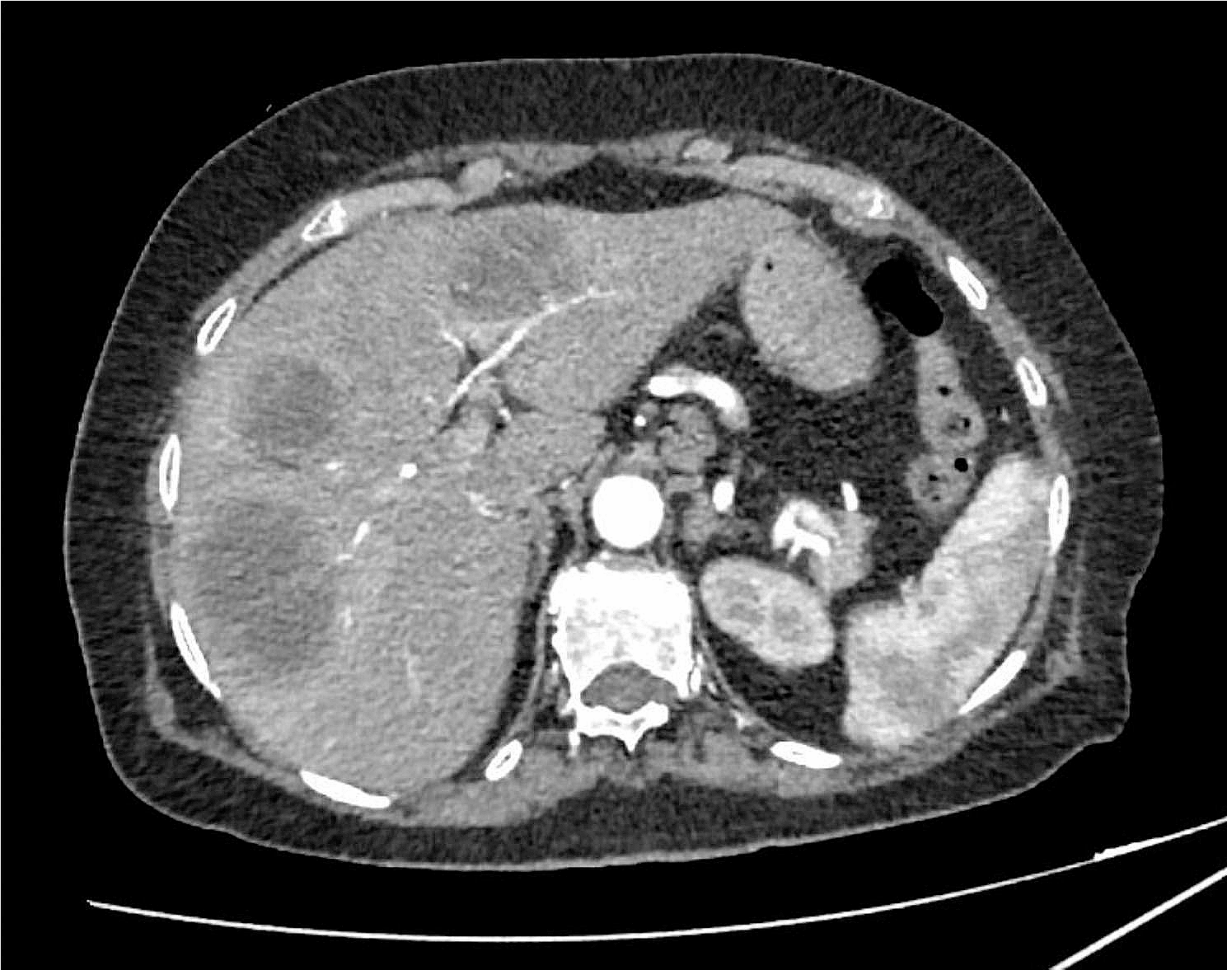
Contrast-enhanced abdominal CT showing the presence, in the liver, of three roundish lesions (SIII-SIV, SV and SV-SVIII) with a cystic morphology. They appear as peripherally enhancing e centrally hypoattenuating lesions; we can also see they are surrounded by an inner ring and they are provided of multiple septa. Due to the peculiar pattern of enhancement, these lesions were considered to be consistent primarily with pyogenic liver abscesses

These lesions appeared as three multiloculated cystic lesions surrounded by pseudocapsules at contrast-enhanced CT scan, suggesting the diagnosis of pyogenic liver abscesses; they were in SIII-IV (4 cm), SV (4 cm) and SV-VIII (8 cm) (Fig. 2).

Though the significant peripheral arterialization indicated they were “pure” abscesses, we also considered whether they hid underlying hepatic metastatic lesions eventually infected by *Sc*. Therefore, we looked for possible primary neoplastic lesion.

Breast examination did not reveal the presence of a palpable mass, and the CT done on day 3rd failed to identify any potential previous unknown thoracic cancer.

A total colonscopy showed a normal mucosa.

Lastly, we focused on the jejunal mass detected at the previous hospitalization. It was no longer observable as a roundish lesion at CT scan; now it appeared as an eccentric thickening (20 × 10 mm) of the same intestinal loop with some inner cystic areas. Whether the lesion was an abscessed intestinal loop, or a group of inflammed diverticula, or the less common Meckel’s diverticulum, the patient underwent a surgical excision of the jejunal mass. Pathology identified the lesion as a Gastrointestinal Stromal Tumour (GIST). Due to its location, size (30 mm) and mitotic index (25/20 HPF), it was considered as high-risk lesion and a chemotherapy with imatinib was then prescribed.

## Discussion and conclusions

The *S. anginosus* group (SGA) consists of three species of Gram positive, catalase negative cocci, named *S. anginosus* (*Sa*), *S. intermedius* (*Si*) and *S. constellatus* (*Sc*). They are nonmotile, facultative anaerobes which can exhibit different haemolytic patterns [2].

Although in general they are part of normal oral and gastrointestinal (GI) flora [3], they can acquire the capacity to give bacteremia and form abscesses. This could be due to the production of both enzymes such as hyaluronidase, which drives the haematogenous spreading in the organism, and of pyrogenic exotoxins such as intermedilysin, which could play a role in the abscesses formation [4].

*Sc* has been increasingly reported as potential determinant of suppurative infections in a variety of sites [5–7] which seem to be related to the primary location of the bacteria and the route of migration. So, liver abscesses are often associated to pathological processes of GI tract (according to venous drainage of this area), which, however, are not necessarily of malignant nature [8, 9]. Blood transmission could also explain other aggressive manifestations related to *Sc*, for example intracranial infectious aneurysms following a pulpitis caused by *Sc*, which was later responsible for an infection in the cavernous sinus [10].

Infection of a metastatic lesion also represent a cause of liver abscesses. Though we ruled out the presence of most common primary colon cancer, eventually we diagnosed a GIST in the jejunum, which may have represented the source of *Sc* bacteremia and liver abscesses.

Only few cases of such an association - *Sc* bacteremia, PLA and GIST - have been reported in the literature [11–15].

More frequently, literature highlighted a relation between SGA and other digestive tract tumours, as esophageal [16], gastric [17, 18] and colorectal cancer [19–22], these last associated to PLA.

In particular, Sasaki H et al. [16, 18] reported cloning of *Sa* DNA fragments from surgical specimens of esophageal and gastric cancers, but not from lung, cervix and kidney ones. Zhou CB et al. [17] found an association between *Sc* as well as *Sa* and gastric cancer and suggested that fecal *Sc* and *Sa* (detected using quantitative PCR) may represent non-invasive accurate signatures for precancerous and early stages of this kind of tumour.

The relationship between *Sc*, PLA and GIST as well as SGA, PLA and colorectal cancer seems similar to the well-described association between these two last entities and *Streptococcus bovis* [23] or – as more recent studies have suggested – *Klebsiella pneumoniae* [24] and even other members of the intestinal microbiota. Some authors consider such relations not causative. They think neoplastic growth can disrupt the integrity of gastro-intestinal barrier, creating a pathway for bacterial translocation and systemic spreading [24].

At the end of our diagnostic work-up, we found a correlation between *Sc*, invasive infections and the presence of a malignancy. Although not yet validate in literature, this relationship should prompt physicians to screen for upper and lower GI neoplasms in patients presenting with sepsis or pyogenic infections caused by *Sc*, as it could facilitate an appropriate and timely treatment.

Further investigation is needed in the future in order to better understand a possible etiopathogenetic role of *Sc* in carcinogenic process or as a potential marker of a GI tumor.

## Data Availability

Not applicable.

## Abbreviations

CRP: C-reactive protein
CT: Computed tomography
ER: Emergency Room
GI: Gastro-intestinal
GIST: Gastro-intestinal stromal tumor
PLA: Pyogenic Liver Abscess
Sc: Streptococcus constellatus
SGA: Streptococcus anginosus group
US: Ultrasound
